# Nutritional behaviour and attitudes in food allergic children

**DOI:** 10.1186/2045-7022-1-S1-O41

**Published:** 2011-08-12

**Authors:** Alice Toniolo, Laura Polloni, Antonella Muraro

**Affiliations:** 1 Veneto Region Food Allergy Referral Centre, Paediatrics, Padua, Italy

## Background

Avoidance of food allergens requires families to adapt dietetic habits, changing nutritional behaviour and attitudes. A restriction of food choice can result in a monotonous diet and impact on social life.

## Objective

We investigated the influence of an elimination diet on behaviour and attitudes of food allergic patients and their families. We explored if age can influence their way of dealing with food allergy.

## Methods

A specific questionnaire was filled-out by 107 patients and their mothers which attend the Food Allergy Centre, Padua, Veneto. The patients (mean age 4.6) were divided into two groups: the first included 72 preschool-children from 0 to 5 years (mean 2.7), while the second group was made-up of 35 children from 6 to 11 (mean 8.46). A descriptive analysis was completed to investigate the nutritional behaviour and their attitude towards food. T-test was used to analyse the differences between the age groups.

## Results

The results showed school-aged children are significantly less interested in tasting new foods (p<.01) than younger children. Most of the children (76;71%) claimed to have a "monotonous" diet; in a rising rating scale from 1 to 5 they reported a mean score of 3.3. No differences were found between the two groups of age. When asked about causes of repetitive diet, the participants answered: strict avoidance (36), difficulties in making traditional recipes (23), a limited choice of food industry safe products (21) and low curiosity about food (23). Regarding participation to social events involving food, 17.5% of older children reported they never attend parties. Those who participate always (22;62.5%), or sometimes (7;20%) eat only "safe foods" (45%), or bring foods from home (24%), or take on both solutions. Younger children answered "sometimes" for the majority (39; 54%), a few "always" (27;37.5%) and only some "never" (6;8.5%).

## Conclusion

The results underline the impact of food allergy in reducing interest about food and in influencing patients’ approach to social life. It is important to support families in arousing curiosity in children, suggesting recipes for a varied and stimulating diet.

